# Probabilistic forecasting of under-five mortality in Uganda: implications for monitoring SDG 3.2

**DOI:** 10.3389/fpubh.2026.1801436

**Published:** 2026-08-13

**Authors:** Rugiranka Tony Gaston, Shaun Ramroop, Faustin Habyarimana

**Affiliations:** School of Agriculture and Sciences, University of KwaZulu-Natal, Pietermaritzburg, South Africa

**Keywords:** ARIMA, probabilistic analysis, SDG 3.2, time-series forecasting, Uganda, under-five mortality

## Abstract

**Background:**

Tracking Sustainable Development Goal (SDG) 3.2 progress requires both explicit quantification of uncertainty and point estimates. Deterministic trend analysis, which has poor predictive power for reaching SDG targets, is the basis for most country-specific estimates of under-five mortality.

**Methods:**

Probabilistic time series forecasting was used to analyse Uganda’s annual under-five mortality data. Forecasts were created using Autoregressive Fractionally Integrated Moving Average (ARFIMA) and Autoregressive Integrated Moving Average (ARIMA) models through 2030. Rolling-origin root mean square error, Taylor diagram and residual diagnostics calibration were used to evaluate predictive accuracy. SDG 3.2-anchored metrics were computed, including the likelihood of achieving these goals by 2030, the absolute gaps that still exist, and the required annual rates of decline.

**Results:**

The ARIMA model predicts sustained declines in under-five mortality based on the available data but remains well above the target of 25 deaths per 1,000 live births set in SDG 3.2 by 2030. The model estimates a mortality gap of about 17.68 deaths per 1,000 live births, corresponding to a 1.98% probability of reaching the SDG target by 2030. Overall, these results suggest that the current rate of decline is insufficient to meet the target, and an average annual reduction of approximately 10.3% is necessary to achieve SDG 3.2. Even though the ARFIMA model showed moderate long-memory dependence, it produced substantially higher forecast errors and less stable long-term projections than the ARIMA model. Consequently, the ARIMA model was identified as the more accurate and reliable approach to predict under-five mortality in Uganda.

**Conclusion:**

Despite realised values falling within anticipated uncertainty limits, Uganda is not on track to meet the SDG 3.2 under-five mortality target for 2030 based on current trends. A clear and practical method for monitoring the SDGs while accounting for uncertainty is probabilistic forecasting.

## 1 Background

In addition to being a crucial indicator of the state of health and the effectiveness of health systems, preventing under-five mortality is still a top global health priority (1). By limiting under-five mortality to 25 deaths per 1,000 live births by 2030, the Sustainable Development Goal (SDG) 3.2 establishes a quantitative standard that may be used to assess country progress monitoring (1, 2). Although under-five mortality has significantly decreased globally during the 1990s, there are still significant subnational and national differences, with many high-risk nations falling short of the SDG target (1, 3). Due to advancements in vaccination, maternal and newborn care, nutrition, and broader socioeconomic transformation, Uganda has seen a notable decline in under-five mortality over the past three decades (2–4). But worries about whether developments are on track for SDG 3.2 throughout the period available have been raised by a slowdown in the rate of advancement in recent years (5–7).

Most existing assessments of SDG progress rely on retrospective descriptions or point projections that do not fully characterise forecast uncertainty or the probability of meeting a specific policy threshold (8, 9). Probabilistic forecasting explicitly accounts for uncertainty in future trajectories and therefore provides more informative evidence for planning and decision-making than deterministic extrapolation (10–12). In public health, probabilistic time-series methods have been applied to infectious disease incidence, mortality, and health service demand, offering calibrated predictive distributions and well-understood diagnostics for model adequacy (11).

Autoregressive Integrated Moving Average (ARIMA) models are a well-established class of univariate forecasting methods widely used in epidemiology and demography for short- to medium-term projections (12, 13). However, demographic time series, including some mortality series can exhibit long-range dependence that is not fully captured by short-memory ARIMA specifications (14, 15). Autoregressive Fractionally Integrated Moving Average (ARFIMA) models generalise ARIMA by allowing fractional differencing, providing a parsimonious way to model long-memory behaviour that may be present in child-mortality trends (16, 17). Comparative studies that examine ARIMA and ARFIMA performance in health forecasting suggest that accounting for long memory can improve point forecasts for some series, but uncertainty quantification and multi-step forecasting require careful implementation and diagnostic checking (15, 18). Forecast evaluation should be performed using out-of-sample rolling-origin approaches and multi-horizon accuracy metrics (such as root mean square error (RMSE) by horizon), combined with probabilistic calibration diagnostics such as probability integral transform (PIT) histograms and formal tests like the Ljung–Box statistic for residual autocorrelation (12, 13, 19–21).

Embedding SDG thresholds directly into probabilistic forecasts such as computing $P(Y_{2030}\leq 25)$ , the projected gap to target, and the constant annual reduction rate required to meet the threshold translates model output into policy-relevant metrics that are immediately interpretable by planners and stakeholders (8, 22). Recent work applying probabilistic and ensemble forecasting approaches to SDG indicators demonstrates the value of explicitly quantifying the likelihood of target attainment and the size of policy interventions required to close gaps (23, 24). In this paper, we apply ARIMA and ARFIMA models to annual under-five mortality data for Uganda from 2000 to 2024, produce probabilistic forecasts to 2030, and derive SDG 3.2 anchored metrics which include the probability of meeting the target, the absolute projected gap, and the required average annual reduction rate. We evaluate model performance using rolling-origin RMSE across multiple horizons and conduct residual diagnostics to ensure model adequacy. By combining time-series forecasting with explicit policy benchmarks, this analysis provides a transparent and decision-relevant assessment of Uganda’s prospects for achieving SDG 3.2. To our knowledge, this is the first study of its kind to compare the ARIMA and ARFIMA probabilistic forecasts of Uganda’s under-five mortality, transforming forecast uncertainty into SDG 3.2 policy metrics, such as the probability of achieving the target, projected mortality gaps, and required annual reduction rates.

## 2 Methods

### 2.1 Data sources

The under-five mortality rate (U5MR) is the probability that a newborn will die before reaching 5 years of age, expressed as the number of deaths per 1,000 live births. Despite being calculated from counts of deaths and live births, it has been reported that U5MR reflects a continuous demographic indicator that has been estimated using harmonised demographic data and statistical modelling techniques. Consequently, the annual U5MR series can be modelled using ARIMA and ARFIMA methods, provided the stationarity assumptions are satisfied. Uganda-level annual U5MR data were retrieved from the World Bank’s World Development Indicators (WDI) database (year range 2000–2024). The study period was chosen to identify Uganda’s trajectory at the MDG 4 period (2000–2015) and the SDG 3.2 period (2016–2030) in order to provide a comprehensive overview of long-term trends in mortality and to determine the trajectory the country is currently on to reach the SDG 3.2 target to reduce under-five mortality to fewer than 25 deaths per 1,000 live births by 2030. The analysis was conducted at the national level using a balanced annual time series for Uganda to preserve temporal mortality trends. The World Bank derives these estimates from harmonised demographic data obtained from civil registration and vital statistics systems, Demographic and Health Surveys (DHS), Multiple Indicator Cluster Surveys (MICS), and model-based adjustments to account for incomplete reporting. Data extraction, preprocessing, and statistical analyses were performed using R version 4.4.0.

### 2.2 Time-series preprocessing

To track long-term trends, persistence, and potential structural abnormalities, the country-specific U5MR time series were visually examined. The Augmented Dickey–Fuller (ADF) test was used to evaluate the null hypothesis of a unit root, while the KPSS test was used to assess the null hypothesis of stationarity (25–27). When necessary, differentiation was used to get rid of the stochastic trend. Care was made to ensure that there was no over differencing in the data, particularly in relation to long-memory behaviour. Residual diagnostic tests were used to assess the adequacy of the model. Residual autocorrelation was assessed using the Ljung–Box test and conditional heteroskedasticity was investigated using the ARCH–LM test. Significant ARCH effects were detected (*p* < 0.01), but no variance model was fitted due to the purpose of the model being point and probabilistic forecasting and not modelling volatility. Prediction intervals were therefore employed to measure forecast uncertainty. Using the auto.arima() function in R’s forecast package, a preliminary identification of ARIMA order was carried out for short-memory models (26, 28). The optimal model is selected using the corrected Akaike Information Criterion (AICc) after this method conducts a stepwise search on candidate AR and MA orders. Forecast accuracy between models was formally compared using multi-horizon RMSE and the Diebold–Mariano test.

### 2.3 Model specification

Suppose that $y_{t}$ denote the annual under-five mortality rate at time t . Two competing time-series frameworks were adopted: ARIMA, which captures short-memory dynamics, and ARFIMA, which accounts for long-term dependence.

#### 2.3.1 ARIMA modelling

The autoregressive integrated moving average ARIMA ( p,d,q ) model is defined as in Equation 1 below:


$$\phi (B){\left(1-B\right)}^{d}y_{t}=\theta (B)Z_{t}$$ (1)

where B is the backshift operator, ϕ(B) and θ(B) are polynomials of orders p and q , respectively, d is the order of differencing, and $Z_{t}$ is a white-noise error term (26, 27). Model selection was guided by AICc minimisation and residual diagnostics, including autocorrelation function (ACF) plots and the Ljung–Box test, to ensure adequacy of fit.

#### 2.3.2 ARFIMA modelling

To capture long-memory behaviour, the ARFIMA ( p,d,q ) model extends ARIMA by allowing the differencing parameter d to take fractional values shown in Equation 2 below:


$$\phi (B){\left(1-B\right)}^{d_{f}}y_{t}=\theta (B)Z_{t},d_{f}\in (-0.5,0.5)$$ (2)

Fractional differencing enables the model to represent slowly decaying autocorrelations commonly observed in demographic and health indicators (14, 27). ARFIMA parameters were estimated using maximum likelihood methods, and model adequacy was assessed using information criteria and residual diagnostics consistent with ARIMA evaluation. Long-memory behaviour is formally assessed using the Geweke–Porter–Hudak (GPH) estimator (27). Residual white-noise behaviour for ARFIMA models was additionally evaluated using Ljung–Box diagnostics in the same manner as ARIMA models. All models were estimated using the appropriately differenced time series, and forecasts were presented on the original under-five mortality scale for interpretation.

### 2.4 Forecast evaluation

Out-of-sample forecast performance was assessed using a rolling forecast horizon of h=1,…,5 years. Forecast accuracy was measured using the root mean squared error (RMSE) given in Equation 3 below:


$$\mathrm{RMS}E_{h}=\sqrt{\frac{1}{n}\sum_{t=1}^{n}{\left(y_{t+h}-{\hat{y}}_{t+h}\right)}^{2}}$$ (3)

Residual autocorrelation was evaluated using the Ljung–Box test, with non-significant *p*-values indicating adequate model specification (26). Comparative performance between ARIMA and ARFIMA models was assessed based on forecast accuracy, residual behaviour, and SDG-aligned indicators.

### 2.5 SDG 3.2 anchored metrics

Forecasts were evaluated relative to the Sustainable Development Goal (SDG) 3.2 target of reducing U5MR to 25 deaths per 1,000 live births by 2030. Three policy-relevant metrics were derived:

1. Absolute SDG gap:
$$\mathrm{GA}P_{2030}={\hat{y}}_{2030}-25$$
2. Required reduction rate:
$$r=1-{\left(\frac{25}{y_{0}}\right)}^{\frac{1}{T}}$$

where $y_{0}$ is the most recent observed under-five mortality rate and T is the number of years to 2030.

Probability of SDG attainment, calculated as shown in Equation 4 below:


$$P({\hat{y}}_{2030}\leq 25)$$ (4)

Probabilities were estimated directly from the predictive distribution of the selected ARIMA model under the assumption of normally distributed forecast errors. The assumption of normally distributed forecast errors was evaluated using residual normal Q–Q plots together with the Shapiro–Wilk and Jarque–Bera normality tests. More precisely, the probability of achieving SDG 3.2 was estimated as the probability that the predicted under-five mortality rate in 2030 will be less than or equal to the target: 25 deaths per 1,000 live births. These estimates provide an interpretable connection between statistically determined forecasts and global child mortality policy targets (27). Forecast uncertainty was quantified using model-based prediction intervals. The forecast standard deviation was derived from the 95% prediction intervals using the normal approximation and is given by Equation 5:


SD=(Upper95−Lower95)/(2×1.96) (5)

## 3 Results

### 3.1 Time-series preprocessing

**Figure 1:**
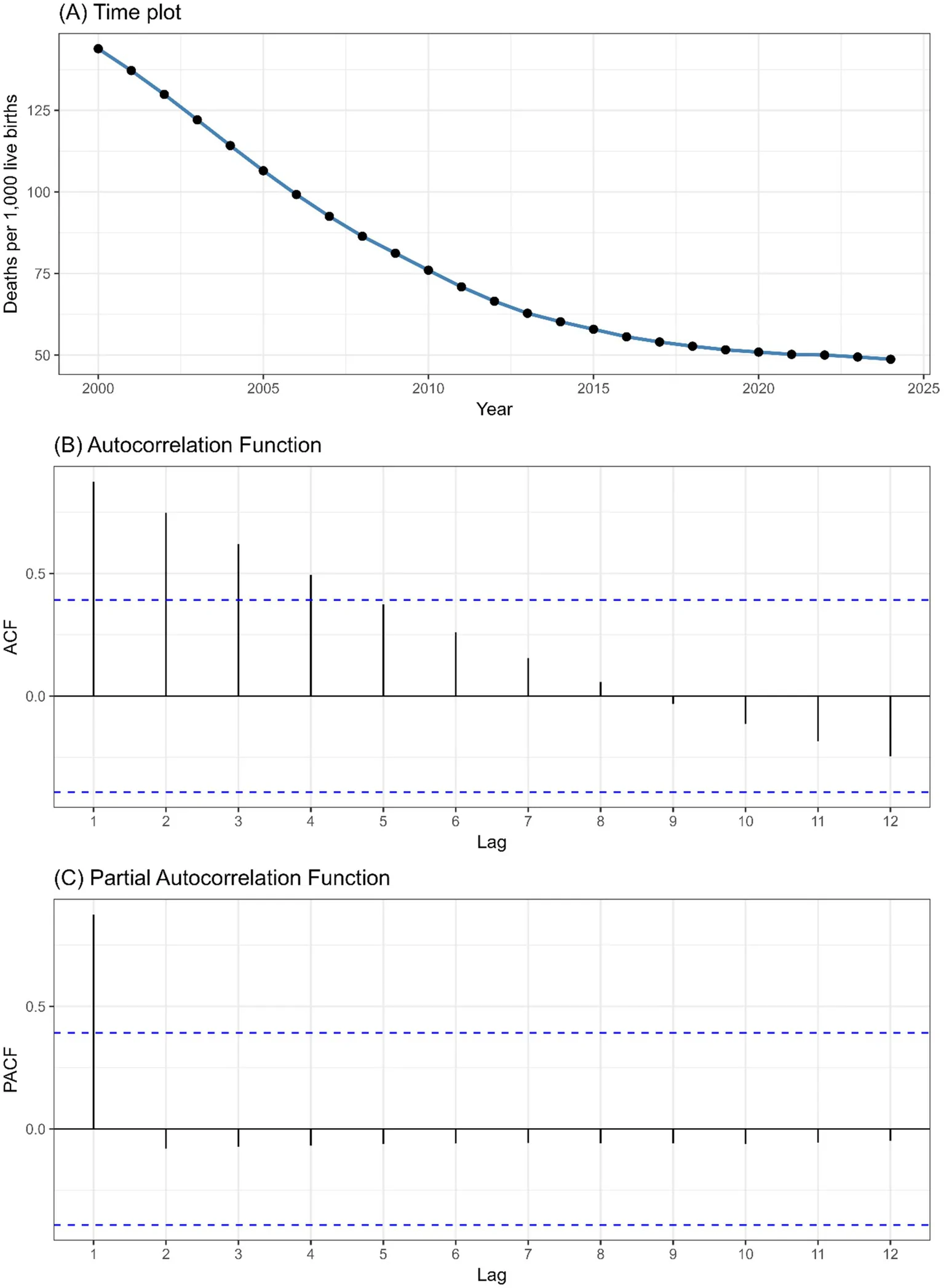
Preliminary time-series diagnostics of annual under-five mortality in Uganda (2000–2024). **(A)** Time plot showing a steady decline in the under-five mortality rate over the study period. **(B)** Autocorrelation function (ACF) indicating significant positive autocorrelations at lower lags that gradually decay with increasing lag, suggesting strong temporal dependence in the series. **(C)** Partial autocorrelation function (PACF) showing a single significant spike at lag 1, with the remaining lags falling within the 95% confidence limits, indicating a low-order autoregressive process.

Figure 1 is a preliminary exploratory investigation for the annual under-five mortality series for Uganda. The time graph (Figure 1A) shows a strong downward trend between 2000 and 2024, indicating that the series’ mean is non-stationary. The autocorrelation function (Figure 1B) displays strong positive autocorrelations which gradually decline over successive lags, suggesting the presence of a trend and substantial temporal dependence. Figure 1C shows a dominant significant spike at lag 1, with the remaining lags falling within the 95% confidence limits. Together, these diagnostic results show that the original series is non-stationary, and differencing is required prior to ARIMA model estimation.

### 3.2 Forecast performance

**Figure 2:**
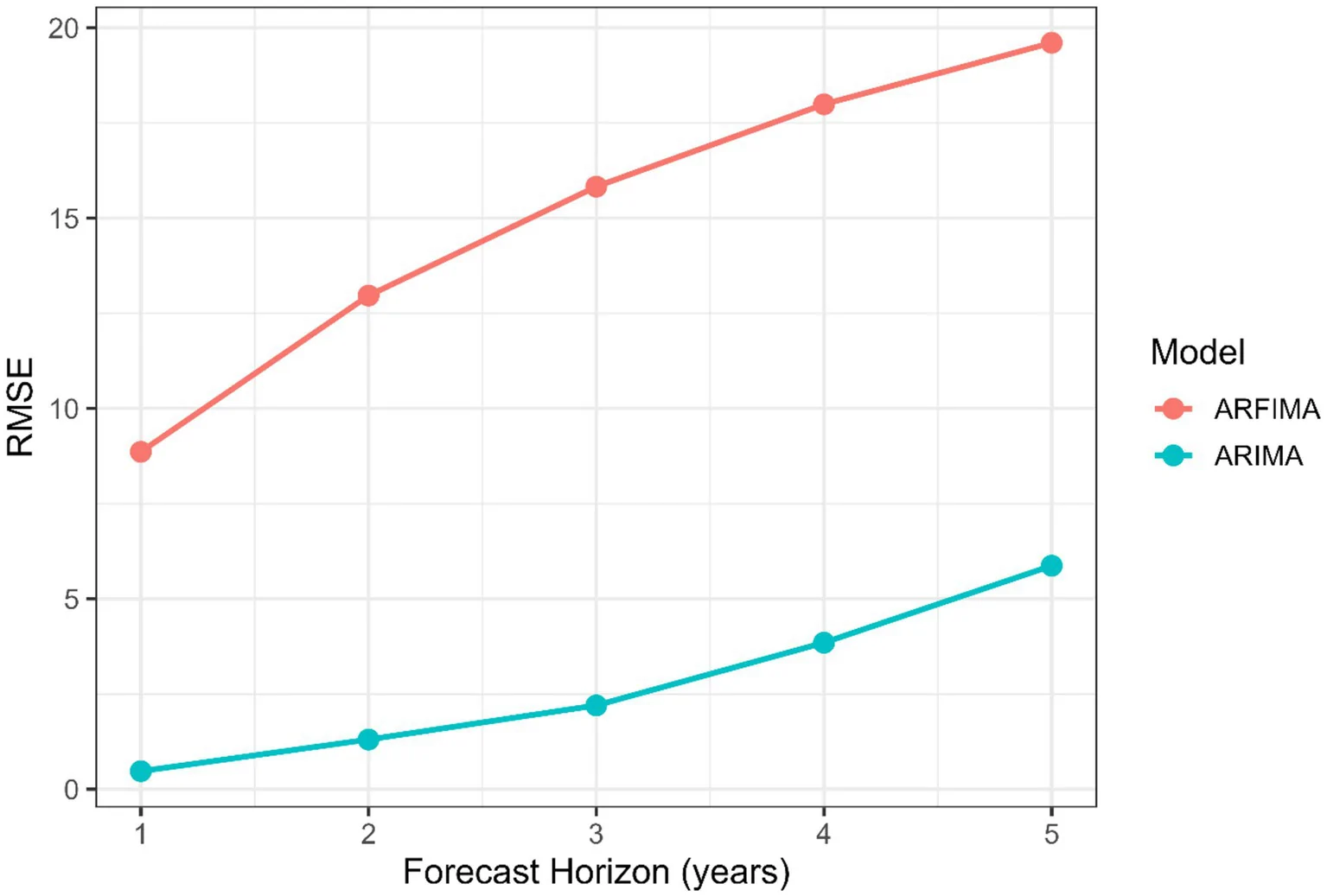
Comparison of multi-step forecasting accuracy for the ARIMA and ARFIMA models across forecast horizons (1–5 years).

Figure 2 compares the multi-step forecasting performance of the ARIMA and ARFIMA models over forecast horizons ranging from 1 to 5 years. For both models, the RMSE increased progressively with the forecast horizon, reflecting the expected accumulation of uncertainty in longer-term predictions. However, the ARIMA model consistently achieved markedly lower RMSE values than the ARFIMA model at every forecast horizon. At the one-year horizon, the ARIMA model produced an RMSE of 0.458 compared with 8.862 for the ARFIMA model. By the fifth-year horizon, the RMSE increased to 5.870 for the ARIMA model and 19.605 for the ARFIMA model. These findings demonstrate that the ARIMA model maintained substantially higher forecasting accuracy and stability across all prediction horizons.

The performance of candidate ARIMA and its ARFIMA models are compared in Table 1. Out of all the ARIMA specifications used, the ARIMA (0, 2, 1) model obtained the lowest AIC (27.99) and BIC (30.17), as well as the smallest forecast errors (RMSE = 0.458; MAE = 0.352), confirming the optimal trade-off between model fit, parsimony, and accuracy. The Ljung-Box test residual diagnostics were non-significant for all the models (*p* > 0.05), indicating that the residuals acted as white noise. While the best performing ARFIMA model estimated a fractional differencing parameter of d = 0.489, showing moderate long-memory dependence, it exhibited substantially higher information criteria and forecast errors than the ARIMA models. Thus, in Uganda the ARIMA (0, 2, 1) model was chosen for forecasting under-five mortality.

**Table 1:** Comparison of ARIMA and ARFIMA models for forecasting under-five mortality in Uganda.

Model	Differencing (d)	AIC	BIC	RMSE	MAE	Ljung–Box *p*-value
ARIMA (0, 2, 1)	**2**	**27.99**	**30.17**	**0.458**	**0.352**	**0.402**
ARIMA (1, 2, 0)	2	28.71	30.89	0.472	0.363	0.289
ARIMA (1, 2, 1)	2	29.93	33.2	0.516	0.409	0.222
ARFIMA (0, 0.489, 0)	0.489	209.11	211.55	8.862	8.107	0.131

AIC, Akaike Information Criterion; BIC, Bayesian Information Criterion; RMSE, Root Mean Square Error; MAE, Mean Absolute Error. Bold indicates the selected final model.

**Figure 3:**
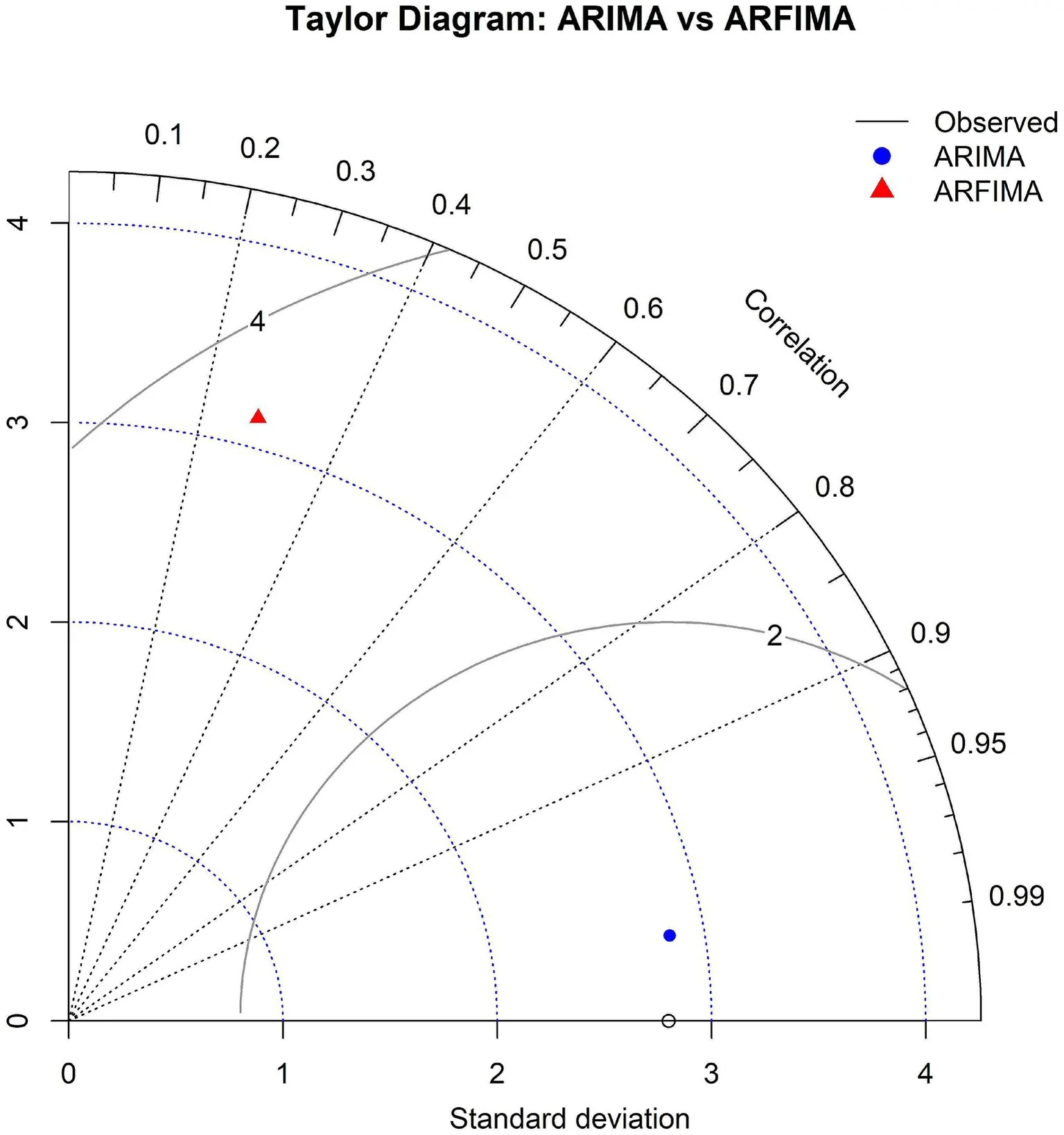
Taylor diagram comparing the predictive performance of the ARIMA and ARFIMA models for Uganda under-five mortality.

Figure 3 shows a Taylor diagram that provides comparisons between the ARIMA and ARFIMA performances in predicting deaths, on which ARIMA lies substantially closer to the reference observation point than ARFIMA, and is better aligned with the observed under-five mortality series. More specifically, the ARIMA model has a higher correlation with the observed data, a standard deviation closer to the observed variability, and a lower centred root mean square error (CRMSE). By contrast, the ARFIMA model has a lower correlation with the observed series, characterised by lower correlation and larger prediction error. Such results serve to visually reinforce the quantitative benchmarking metrics presented in Tables 1, 2, again validating the advantages of the ARIMA model to predict under-five mortality in Uganda.

**Table 2:** Comparison of ARIMA and ARFIMA models for forecasting under-five mortality in Uganda.

Metric	ARIMA model	ARFIMA model
Model specification (*p*, *d*, *q*)	**(0, 2, 1)**	(0, 0.489, 0)
Projected U5MR in 2030	**42.68**	63.78
Probability of achieving SDG 3.2 by 2030 (%)	**1.98%**	3.20%
Projected mortality gap from SDG target in 2030 (deaths per 1,000 live births)	**17.68**	38.783
Required annual reduction rate to achieve SDG 3.2 (%)	**10.30%**	10.30%
RMSE (1-step ahead)	**0.474**	8.862
RMSE (2-step ahead)	**1.305**	12.965
RMSE (3-step ahead)	**2.201**	15.827
RMSE (4-step ahead)	**3.849**	17.990
RMSE (5-step ahead)	**5.870**	19.605
Ljung Box test p-value	**0.402**	0.131

U5MR, under-five mortality rate; SDG, Sustainable Development Goal; RMSE, Root Mean Square Error. Bold indicates the selected forecasting model (ARIMA), which demonstrated superior overall forecasting performance.

Table 2 provides an examination of ARIMA and ARFIMA forecasting performance. ARIMA (0, 2, 1) consistently provided significantly lower RMSE values and acceptable residual diagnostics compared to ARFIMA (0, 0.489, 0) across all forecast horizons (Ljung–Box test, *p* = 0.402), indicating that no significant residual autocorrelation was observed. Despite the estimated fractional differencing parameter d = 0.489 by the ARFIMA model, hinting at modest long-memory interdependence, this prediction improvement was not observed. Each of the models estimated a very low risk of achieving the SDG 3.2 target in Uganda by 2030, with ARIMA and ARFIMA estimating probabilities of 1.98 and 3.20%, respectively. Also, for each model, an average annual reduction of about 10.3% in under-five mortality would be required to meet the SDG target by 2030. The ARFIMA model provided a marginally higher estimated probability for the SDG 3.2 target by 2030 (3.20% vs. ARIMA 1.98%), although this was due more to the larger uncertainty of its predictive distribution than any more favourable expected direction towards mortality. The ARFIMA point forecast was still much further off from the SDG target in relation to the corresponding ARIMA outcome.

**Figure 4:**
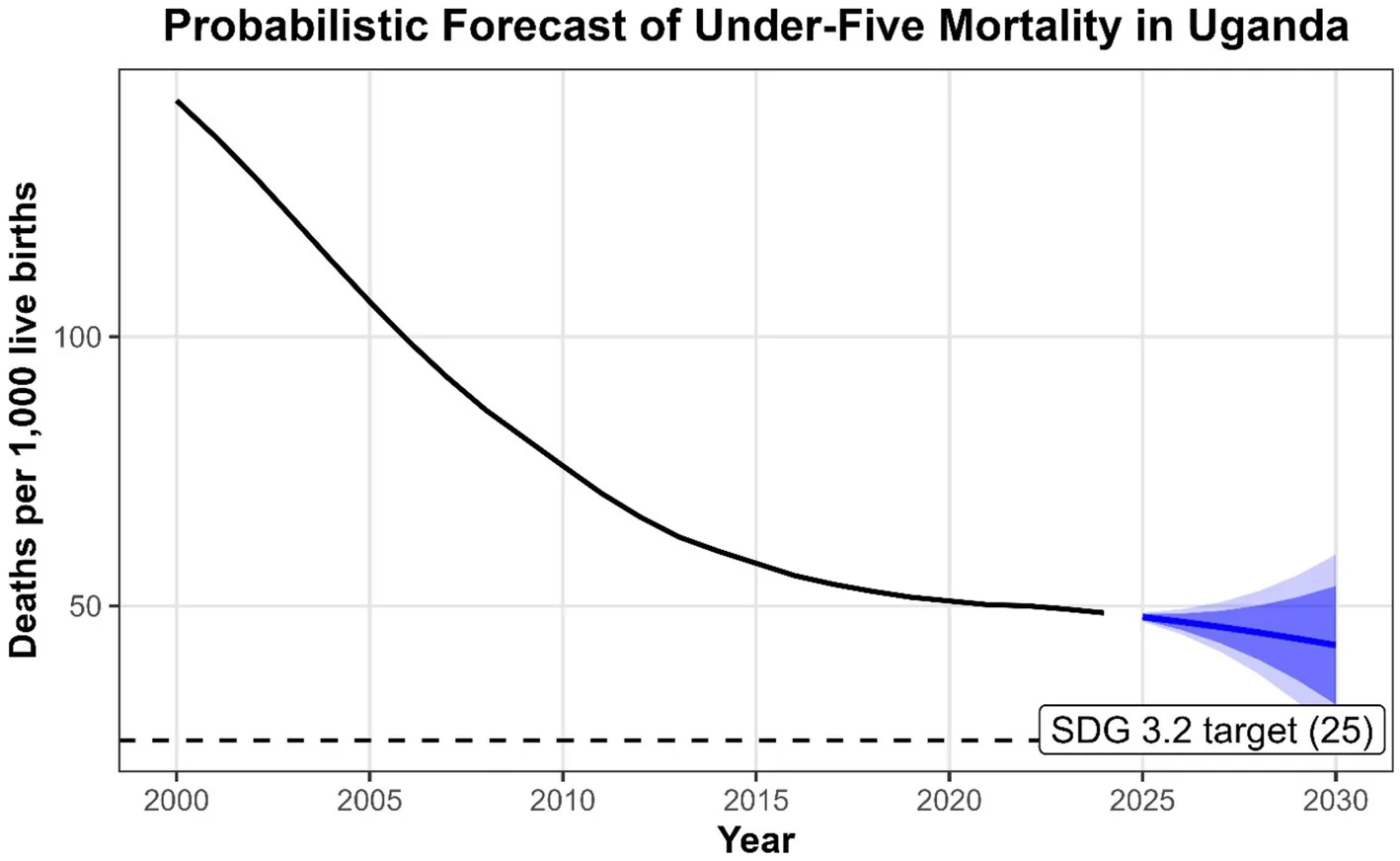
Probabilistic forecast of under-five mortality in Uganda (2000–2030).

Figure 4 depicts the probabilistic ARIMA forecast for Uganda’s under-five mortality rate from 2025 to 2030. The point forecast indicates a continuing gradual decrease in mortality over the forecast period. As the forecast advances further into the future, the 80 and 95% prediction intervals widen, indicating increasing uncertainty. Even with a downward mean forecast, both prediction intervals remain predominantly above the SDG 3.2 target of 25 deaths per 1,000 live births by 2030, indicating that Uganda is unlikely to attain the target under the projected trend without more rapid child survival improvement.

### 3.3 Probabilistic SDG assessments

**Figure 5:**
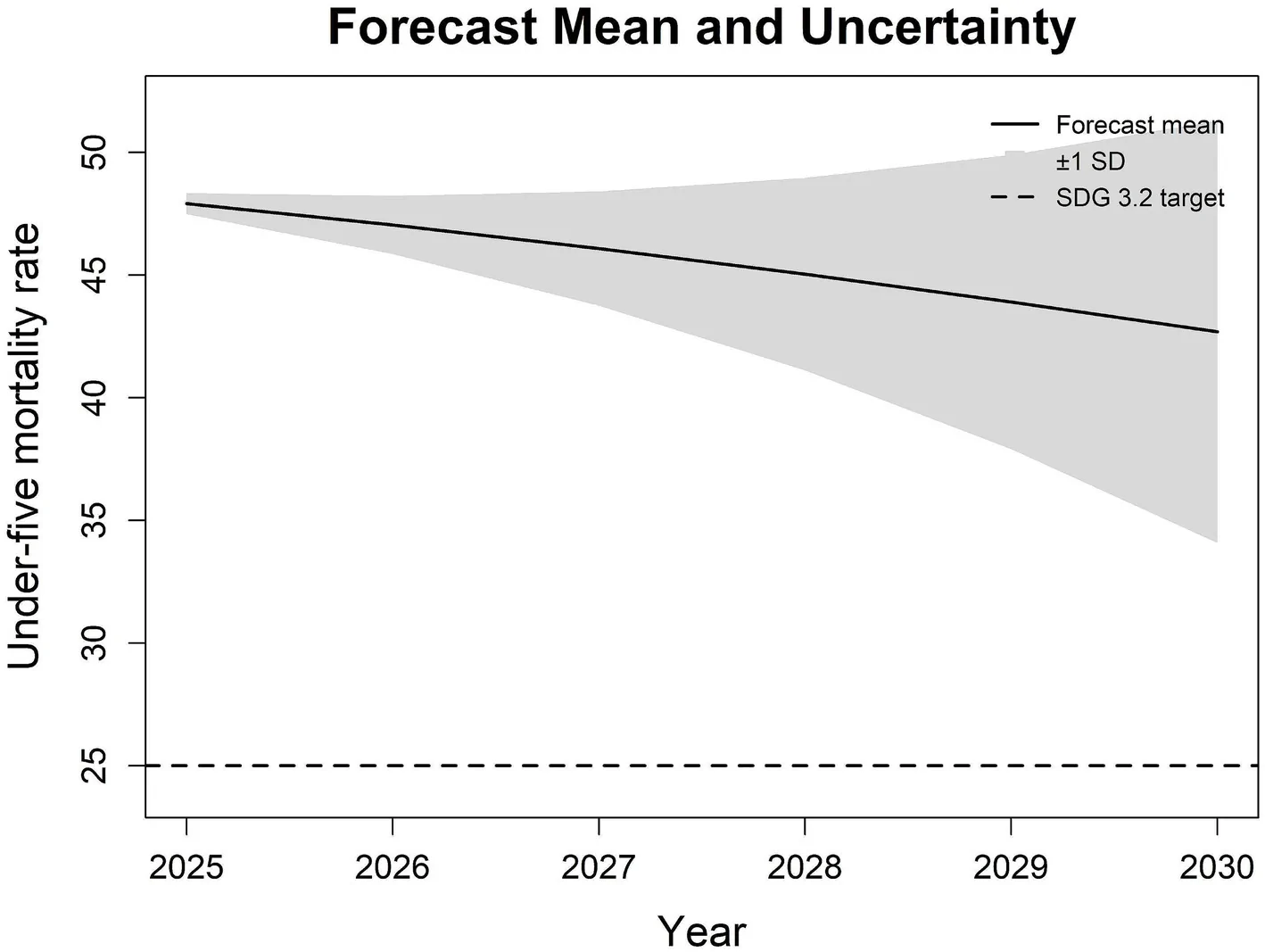
Mean forecast and uncertainty of the under-five mortality rate in Uganda for the period 2025–2030.

Figure 5 presents the projected mean under-five mortality rate in Uganda together with forecast uncertainty for 2025–2030. According to the ARIMA model, the number of deaths per 1,000 live births decreases progressively from approximately 48 in 2025 to about 43 deaths per 1,000 live births by 2030. The variability of the prediction line widens with time, as shown by the widening forecast area. Even though this declines, the entire uncertainty interval remains above the SDG 3.2 target of 25 deaths per 1,000 live births by 2030, implying that Uganda is unlikely to meet the goal given its present trajectory.

**Figure 6:**
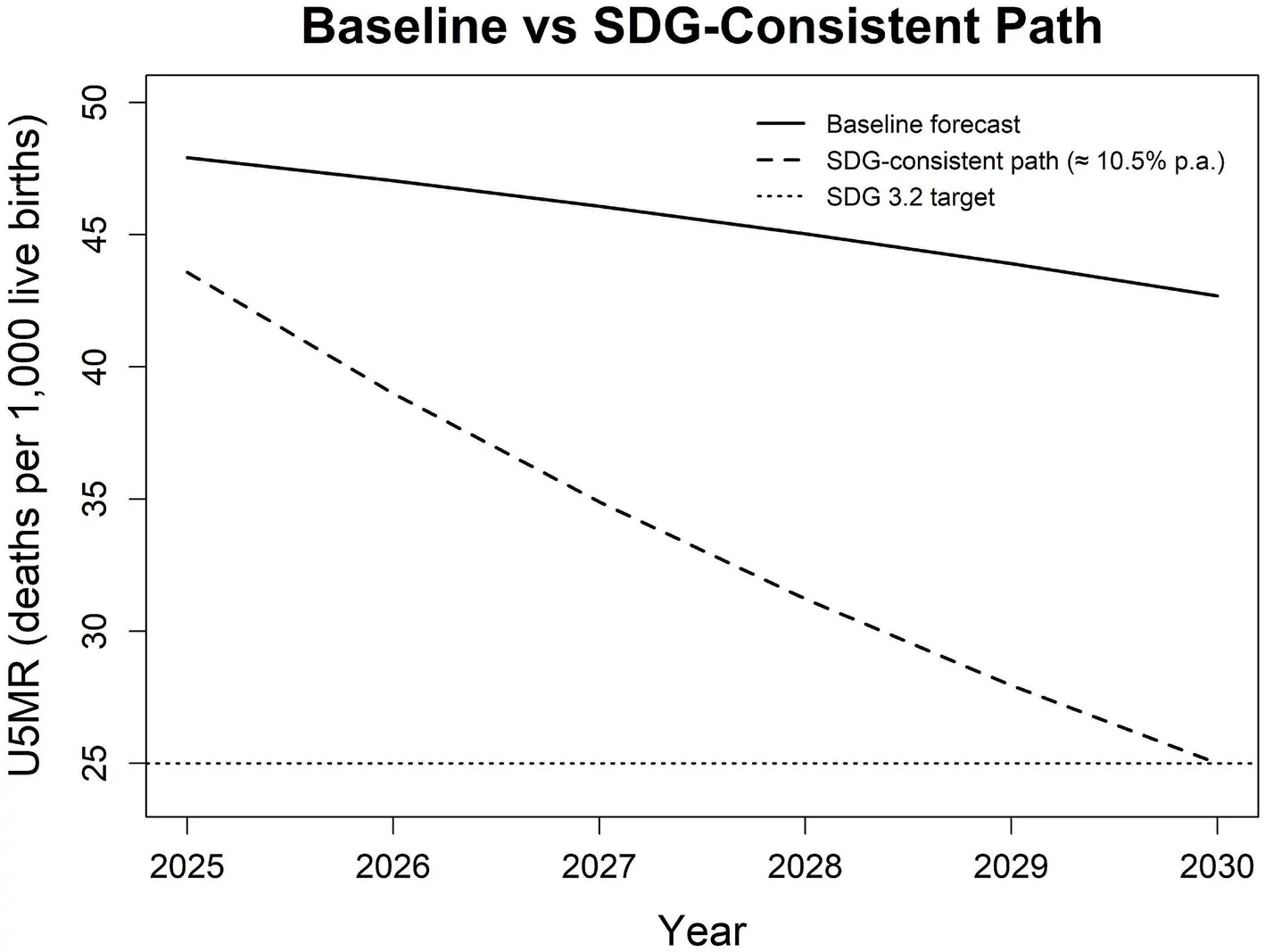
Comparison of the baseline ARIMA forecast and the SDG-consistent trajectory for under-five mortality in Uganda (2025–2030).

Figure 6 compares the baseline ARIMA forecast to the mortality trajectory expected for Uganda to achieve SDG 3.2 by 2030. The baseline forecast predicts a slow decrease in under-five mortality from around 48 deaths per 1,000 live births in 2025 to about 43 deaths per 1,000 live births by 2030. The SDG target would, by contrast, be reached only if that decline became significantly stronger, at an average annual decrease of approximately 10.3%. The increasing gap between the projected and SDG-consistent trajectories further suggests that Uganda is unlikely to achieve the target by 2030 without substantial acceleration in child survival intervention.

### 3.4 ARIMA against ARFIMA comparison

**Figure 7:**
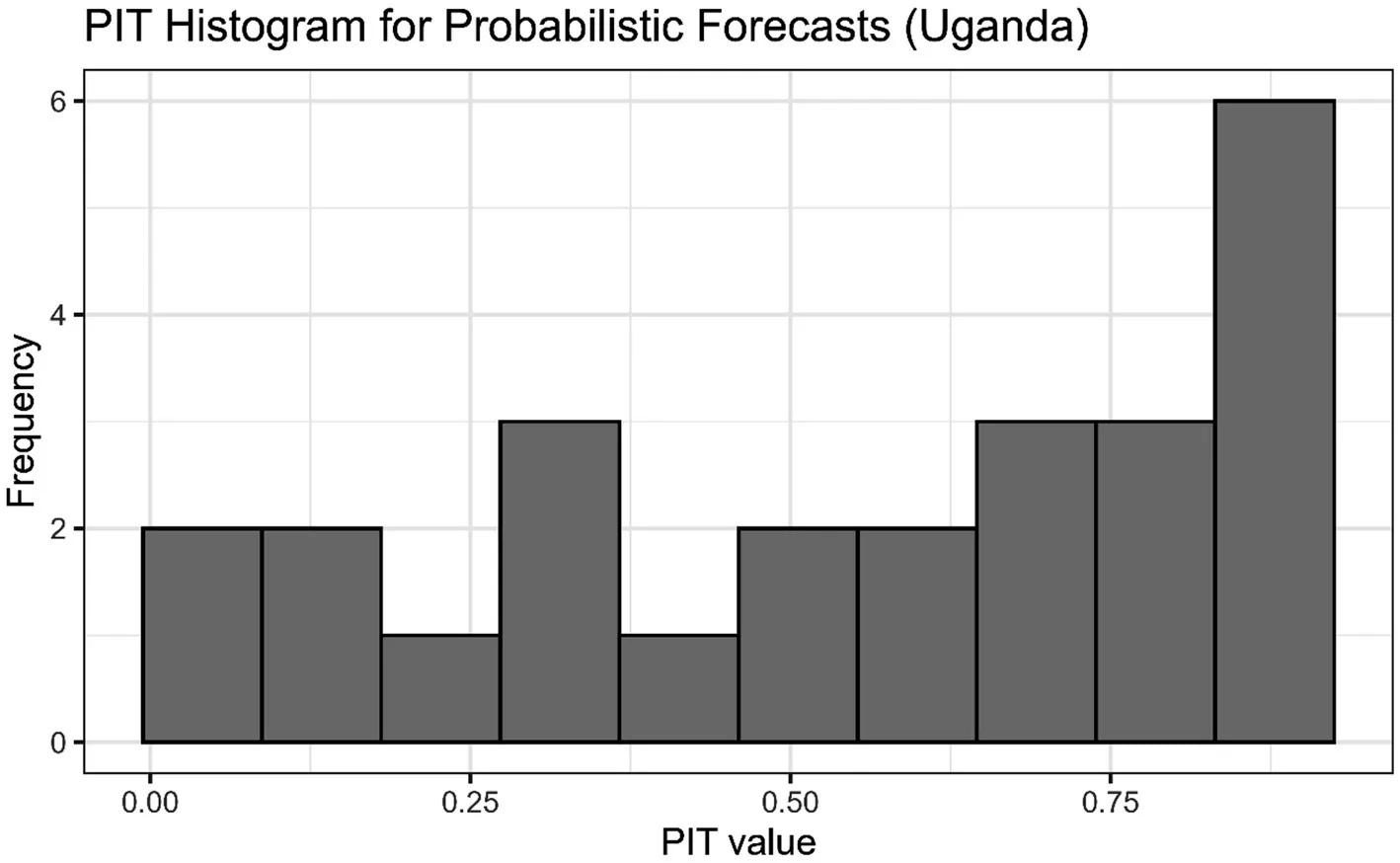
Probability Integral Transform (PIT) histogram for the probabilistic forecasts of under-five mortality in Uganda.

For a fitted ARFIMA model, the fractional differencing parameter d = 0.488 suggests moderate long-memory dependence while remaining within the stationary region. However, despite this evidence of persistence, ARIMA consistently performed better than ARFIMA with reduced forecast errors (RMSE and MAE), better model fit according to AIC and BIC, more stable probabilistic forecasts, and well-behaved prediction intervals. The results indicate that introduction of long-memory behaviour through fractional differencing did not produce better forecasts for the under-five mortality series. The substantially larger projected SDG gap from the ARFIMA model reflects its higher long-term forecast level rather than superior predictive performance, as demonstrated by its consistently larger forecast errors and poorer out-of-sample validation. Thus, the ARIMA model was chosen as the preferred model to forecast under-five mortality in Uganda. Figure 7 illustrates the Probability Integral Transform (PIT) histogram that is used to assess the calibration of the probabilistic forecasts. A perfectly calibrated forecasting model produces PIT values that are approximately uniformly distributed between 0 and 1. The histogram exhibits a noticeable concentration of values in the upper range of the distribution, suggesting that the forecasts may exhibit some degree of miscalibration. Specifically, the accumulation of PIT values near one indicates that the observed mortality rates were frequently lower than anticipated by the predictive distributions, implying a tendency for the model to overestimate future under-five mortality or underestimate the rate of decline.

**Figure 8:**
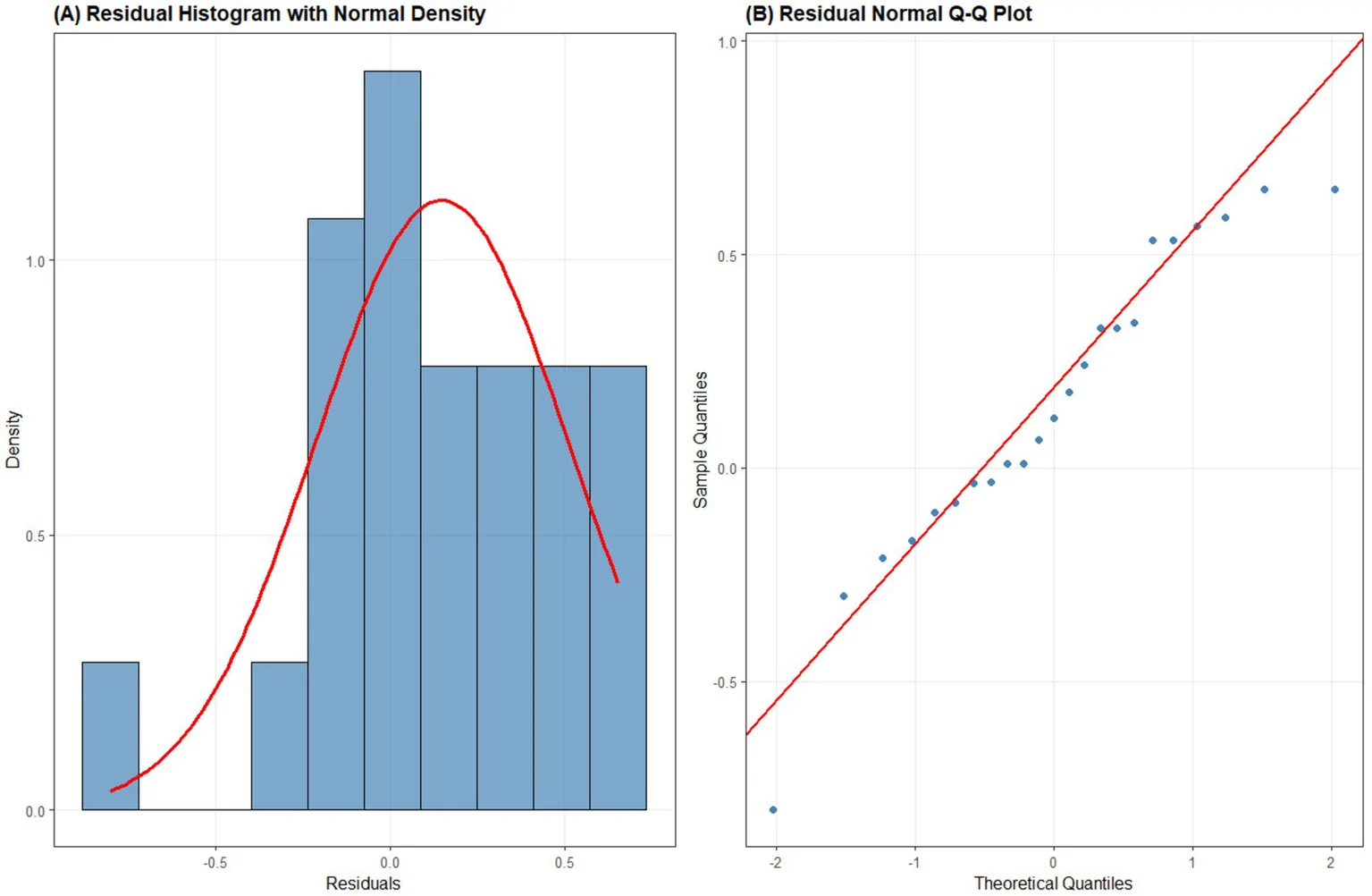
Residual normality diagnostics for the fitted ARIMA model. **(A)** Histogram of the model residuals with the fitted normal density curve (red), illustrating the distribution of the forecast errors. **(B)** Normal Q–Q plot of the residuals comparing the observed.

Normality diagnostics of fitted ARIMA model residuals are shown in Figure 8. The residual histogram (Figure 8A) is roughly bell shaped and centred around zero, and the empirical distribution follows the fitted normal density curve closely. Slight asymmetry is noted but no material deviations from normality are found. The normal Q–Q plot (Figure 8B) shows that the residuals lie close to the reference line, with only minor deviations at the lower and upper tails. These tail departures are natural for small annual time-series and do not indicate serious violations of the normality assumption. This is evidenced by the Shapiro–Wilk test (W = 0.94, *p* = 0.20) and Jarque–Bera test (χ^2^ = 1.10, *p* = 0.60) which did not reject the null hypothesis of normality. Overall, the graphical and statistical diagnostics suggest that the residuals are approximately normally distributed, supporting the use of the normal approximation for constructing prediction intervals and probabilistic forecasting related to SDG 3.2 and to confirm the capability of the fitted ARIMA model for statistical inference and forecasting.

**Figure 9:**
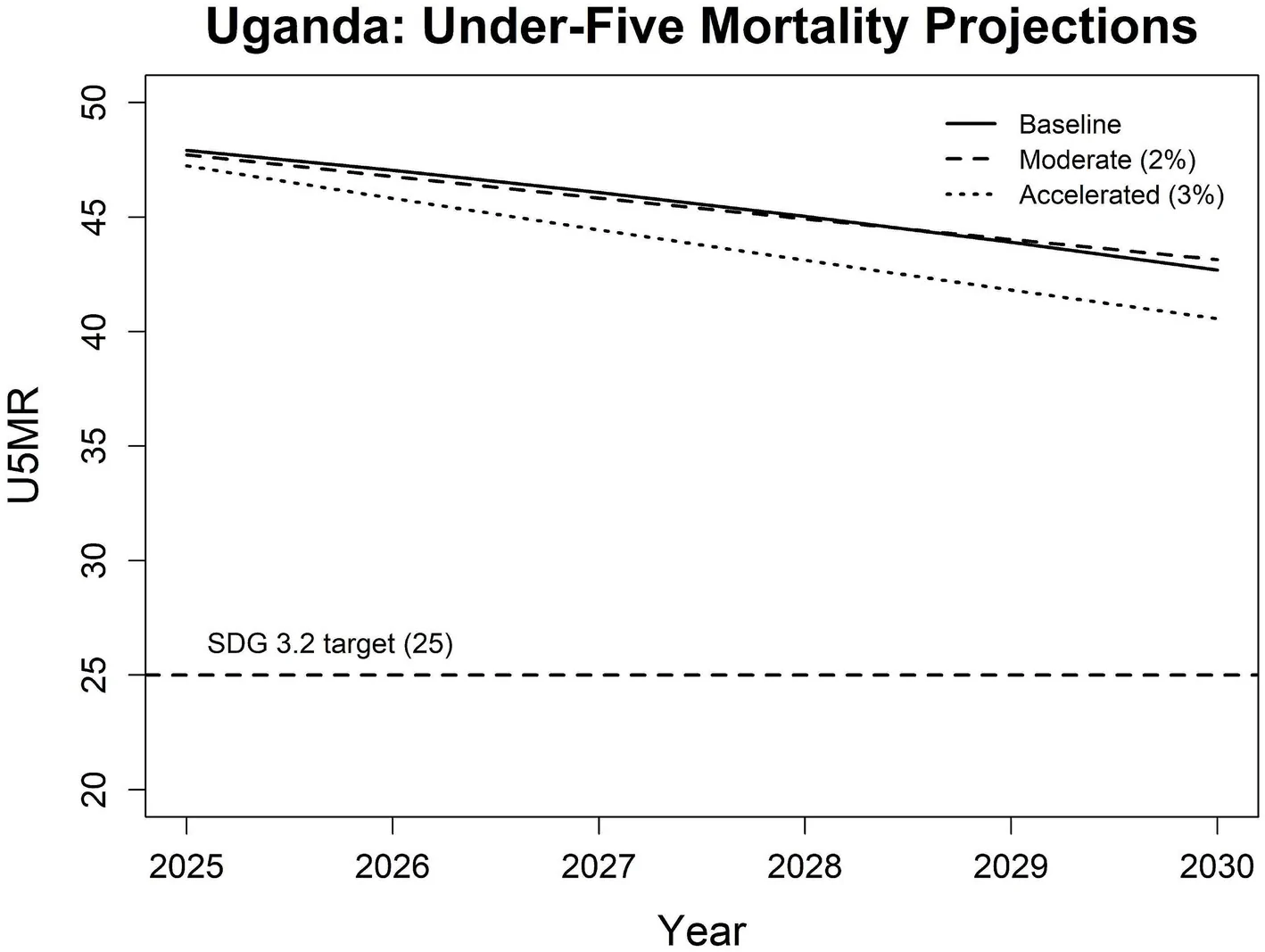
Projected under-five mortality rate in Uganda under alternative reduction scenarios (2025–2030). The solid line represents the baseline ARIMA forecast, while the dashed and dotted lines represent hypothetical moderate (2%) and accelerated (3%) annual reduction scenarios. The horizontal dashed line indicates the SDG 3.2 target of 25 deaths per 1,000 live births.

Figure 9 shows a comparison of the baseline ARIMA forecast and two hypothetical intervention scenarios that represent moderate (2% annual reduction) and accelerated (3% annual reduction) reductions in under-five mortality. The baseline forecast predicts a steady decline in the mortality rate from about 48 deaths per 1,000 live births in 2025 to about 43 deaths per 1,000 live births by 2030. The moderate and accelerated scenarios lead to progressively lower mortality rates over the forecast horizon, but neither scenario achieves the target of 25 deaths per 1,000 live births by 2030 specified in SDG 3.2. The implication is that relatively modest yearly progress would simply be insufficient to meet the international target.

## 4 Discussion

The main contribution of this study is the application of ARIMA and ARFIMA models within a probabilistic forecasting framework to estimate under-five mortality in Uganda and progress on Sustainable Development Goal (SDG) 3.2 (10, 11). However, it has been shown that while under-five mortality is expected to persist in declining under prevailing trends, the mortality rate will stay well above the SDG target of 25 deaths per 1,000 live births by 2030. The selected ARIMA model estimated only a 1.98% probability of Uganda achieving the SDG 3.2 target by 2030 under current mortality trends. The scenario analysis further showed that much faster annual reductions would be needed to close the projected gap and achieve SDG 3.2 (2).

From a methodological perspective, the ARIMA (0, 2, 1) model consistently fared better than the competing models. The pronounced non-stationary trend evident in the mortality series was effectively addressed through second-order differencing, resulting in lower information criteria, smaller forecast errors, and satisfactory residual diagnostics (26–28). While the fractional differencing parameter d = 0.489 for the ARFIMA model reflected moderate long-memory dependence, this increased complexity did not lead to superior prediction. Instead, the ARFIMA model generated substantially larger forecast errors, poorer model fit, and less stable long-term predictions for all forecast horizons. Such results are in keeping with earlier research (14, 15, 29) that showed fractional differencing does not necessarily improve forecasting performance when the dominant feature of the series is a strong deterministic trend rather than persistent stochastic dependence.

An important methodological contribution of this study is the integration of probabilistic forecasting into SDG monitoring. Unlike conventional deterministic projections commonly reported in the child mortality literature, the proposed framework explicitly quantifies forecast uncertainty through prediction intervals and estimates the probability of achieving the SDG target (10, 11). Furthermore, the incorporation of calibration diagnostics, including the Probability Integral Transform (PIT) histogram, Taylor diagram, and multi-step forecast evaluation, provides a comprehensive assessment of predictive performance beyond conventional point forecast accuracy measures (12, 13). These complementary diagnostics improve confidence in the reliability and interpretability of probabilistic forecasts for health policy applications.

The projected reductions in under-five mortality are consistent with those that have been released by the United Nations Inter-Agency Group for Child Mortality Estimation, which have reported that worldwide and sub-Saharan Africa have recorded sustained reductions in child mortality over recent decades (3, 5). These trends have been observed in national-scale analyses from Ethiopia and Ghana, where great progress has been coupled with persistent uncertainty about SDG 3.2 achievement (6, 7). Probabilistic instead of deterministic forecasting is in line with recent recommendations in epidemiological and public health forecasting which emphasise the need to quantify uncertainty explicitly for evidence-based decisions (8).

The comparison of ARIMA with ARFIMA models also adds a significant contribution to the general forecasting literature. While long-memory models have been able to achieve better predictive efficiency for certain applications, their superiority is not universal (16, 17). Meanwhile, in the current investigation, the moderate persistence discovered by the ARFIMA model was not enough to compensate for the higher model complexity, and the forecasting performance was markedly poorer than that based on the simpler ARIMA model. Support for this trend can be seen in the previous studies which indicate that ARIMA models are still a very good predictor of demographic characteristics characterised primarily by long-term trends rather than strong long-memory dependency (14, 15, 27, 28).

From a policy perspective, probabilistic SDG analysis gives more information than point forecasts, especially in complex health systems that are uncertain and exposed to external shocks (1, 2). The low probability of achieving SDG 3.2 under current trends suggests that incremental improvements alone will be insufficient. Instead, accelerated investments in maternal and child healthcare, immunisation programmes, nutrition, water and sanitation services, and broader health system strengthening will be required to substantially reduce under-five mortality (9, 22). The proposed framework provides policymakers a transparent evidence basis for prioritising interventions and tracking progress towards the 2030 Agenda for Sustainable Development by establishing both forecast uncertainty and the prospect of accomplishing international goals on a quantitative basis (2). Uganda has made significant progress in reducing under-five mortality by expanding immunisation, malaria control, HIV prevention, and maternal health programmes in about two decades. Yet persistent regional variations, a shortage of trained health professionals, neonatal mortality, and inequitable access to health care keep the process from moving further. These structural obstacles may contribute to this pattern to explain why existing trends in deaths in this area still fall short of fulfilling the criteria for the attainment of SDG 3.2 by 2030.

A major strength of this study is that it integrates probabilistic forecasting, formal calibration diagnostics, multi-horizon forecast evaluation, and policy-oriented scenario analysis into a single analytical framework (10, 21). By directly connecting statistical projections to the likelihood of achieving SDG 3.2, the study goes beyond conventional mortality predictions from just providing point estimates and offers a more informative context for evidence-based planning. However, a few limitations need to be acknowledged. The estimation used annual national-level mortality estimates and did not explicitly incorporate socioeconomic level, health interventions, environment type or spatial diversity, which might affect child mortality (23, 24, 30). As the World Bank WDI series comprises harmonised demographic estimates rather than direct observations, the present study forecasts an already modelled mortality trajectory. Consequently, uncertainty arising from the underlying estimation process is not explicitly propagated into the forecasting model, and the forecasts should therefore be interpreted as projections of the WDI estimates rather than raw mortality counts. Future studies could compare these findings with estimates from the United Nations Inter-agency Group for Child Mortality Estimation (UN IGME). Also, while our chosen ARIMA model offered acceptable diagnostic accuracy, uncertainty naturally becomes higher with longer predictions and consequently need to be treated with caution when interpreting long-term forecasts (19, 20, 29). To improve future estimates, further studies might integrate explanatory variables using dynamic regression or Bayesian time-series models, supplement the report with sub-national or spatial mortality data, and apply hybrid statistical and machine learning methods to enhance long-range forecasting accuracy and strengthen monitoring of SDGs.

Supporting the target of SDG 3.2 in Uganda will also be in the existing child survival programmes such as Expanded Programme on Immunisation (EPI), Integrated Child Health Days, malaria prevention and control initiatives, and improved neonatal care services. Strengthening the capacity of Community Health Extension Workers to enhance early diagnosis, referral, and community-based maternal and child health services may also help to speed up efforts to reduce under-five mortality. Targeted interventions will help to convert these probabilistic forecasts into actionable strategies to improve child survival, especially in underserved and high-burden regions.

## 5 Conclusion

This study applied ARIMA and ARFIMA models within a probabilistic forecasting framework to evaluate under-five mortality trends and Uganda’s progress towards Sustainable Development Goal (SDG) 3.2. The analysis shows an overall downward trend in under-five mortality, but a trajectory significantly above the SDG target of 25 deaths per 1,000 live births by 2030. The ARIMA model used suggests only a 1.98% probability of reaching the SDG target under current trends, emphasising the requirement of more and faster interventions to enhance child survival.

Comparative model evaluation indicated that the ARIMA (0, 2, 1) model consistently outperformed the ARFIMA model, achieving lower AIC and BIC values, smaller forecast errors, and more satisfactory diagnostic performance. Despite moderate long-memory dependence detected in the ARFIMA model, fractional differencing did not improve forecasting accuracy or long-term predictive stability for the annual under-five mortality series. Therefore, the ARIMA model is the more appropriate and parsimonious method for forecasting under-five mortality in Uganda.

Methodologically, this study extends traditional child mortality forecasting by integrating probabilistic forecasting, prediction intervals, calibration diagnostics, multi-horizon forecast evaluation, and scenario-based SDG assessment within a single analytical framework. By providing a unifying analytical framework for predictive methods rather than mere point predictions, and quantifying forecast uncertainty and estimating the probability of attaining SDG 3.2, the present methodology serves as a more informed framework for evidence-based health planning and policy evaluation than traditional deterministic point forecasts.

Future studies should expand this framework to include covariates from socioeconomic, demographic, environmental, and health system perspectives in future research by using dynamic regression, Bayesian, or spatial–temporal modelling techniques. Testing hybrid statistical and machine learning forecasting models may enhance long-term predictive accuracy and facilitate more adaptable, empirical monitoring of progress towards SDG 3.2. Such advancements would augment the methodological base of child mortality forecasting and offer policymakers more powerful evidence to inform timely interventions to reduce under-five mortality.

## Data Availability

The under-five mortality data analysed in this study are publicly available from the World Bank World Development Indicators (WDI) database. The cleaned analytical dataset and R scripts used to generate the results are available from the corresponding author upon reasonable request.
